# Efficacy of H_2_O_2_ inactivated bovine virus diarrhoea virus (BVDV) type 1 vaccine in mice

**DOI:** 10.1186/s12917-024-03897-0

**Published:** 2024-02-03

**Authors:** Cunyuan Li, Jinming Yu, Yue Wang, Xiaoyue Li, Yaxin Li, Mingxuan An, Wei Ni, Kaiping Liu, Shengwei Hu

**Affiliations:** College of Life Sciences, Shihezi University, Shihezi, 832003 Xinjiang China

**Keywords:** BVDV type 1, H_2_O_2_ inactivated vaccine, Mice, IgG, Cytokines

## Abstract

**Background:**

Bovine viral diarrhea (BVD) is an acute febrile infectious disease caused by the bovine viral diarrhea virus (BVDV), which has brought huge economic losses to the world's cattle industry. At present, commercial inactivated BVDV vaccines may cause some adverse reactions during use. This study aims to develop a safer and more efficient inactivated BVDV vaccine.

**Methods:**

Here, we described the generation and preclinical efficacy of a hydrogen peroxide (H_2_O_2_) inactivated BVDV type 1 vaccine in mice, and administered it separately with commercial vaccine (formaldehyde inactivated) in mice to study its efficacy.

**Results:**

The BVDV type 1 IgG, IFN- γ, IL-4 and neutralizing antibody in the serum of the H_2_O_2_ inactivated vaccine group can be maintained in mice for 70 days. The IgG level reached its maximum value of 0.67 on the 42nd day, significantly higher than the commercial formaldehyde inactivated BVDV type 1 vaccine. IFN- γ and IL-4 reached their maximum values on the 28th day after immunization, at 123.16 pg/ml and 143.80 pg/ml, respectively, slightly higher than commercial vaccines, but the effect was not significant. At the same time the BVDV—1 neutralizing antibody titer reached a maximum of 12 Nu on the 42nd day post vaccination.

**Conclusions:**

The H_2_O_2_ inactivated BVDV vaccine has good safety and immunogenicity, which provides a potential solution for the further development of an efficient and safe BVDV vaccine.

**Supplementary Information:**

The online version contains supplementary material available at 10.1186/s12917-024-03897-0.

## Introduction

Bovine viral diarrhea virus (BVDV) belongs to the genus *Pestivirus* of the family *Flaviviridae* [1, 2]. It is a positive sense ribonucleic acid (RNA) virus with a genome of 12.5 kb and is the cause of bovine viral diarrhea (BVD) [3]. In the 1940s, this virus was first discovered in a diseased cow with peptic ulcer and diarrhea [4]. The onset of BVD is usually about one month, and it is about 2%-50%. However, the mortality rate of cattle infected with the virus is 90% [5]. Generally, cattle of different ages have different clinical symptoms of BVDV infection, it does the most damage to calves [6, 7]. BVDV can also infect the fetus through the placenta and cause persistent infection in calves [8]. BVDV has caused huge economic losses to the livestock [5, 9]. Therefore, it is of great significance to develop a safe and efficient BVDV vaccine for the prevention of BVDV.

Formaldehyde is the most commonly used inactivating agent in vaccine production, but research had shown that formaldehyde-inactivated respiratory syncytial virus (RSV) vaccines have serious complications [10]. Formaldehyde can also destroy key neutralizing epitopes, leading to worsening diseases [11]. Inactivation of the virus with β-propiolactone (βPL) may also induce adverse immune reactions and has been shown to have carcinogenic effects in animals [12, 13]. Hydrogen peroxide (H_2_O_2_) is a highly effective oxidant, often used as an antibacterial agent and preservative [14]. It can destroy the lipid membrane bilayer, oxidize the protein backbone and cause oxidative damage to nucleic acids [15]. In addition, H_2_O_2_ can be decomposed into water and oxygen and discharged from the air treatment system, which means that it does not require post-processing, or neutralization and leave residues, making H_2_O_2_ more environmentally friendly [16]. Research had shown that influenza viruses inactivated with H_2_O_2_ not only retain immunogenicity but also trigger humoral and cellular immune responses [17]. Abd-Elghaffar et al. proved that H_2_O_2_ can irreversibly inactivate the rabies virus without affecting its antigenicity and immunogenicity [18]. Furthermore, the inactivation of microorganisms with H_2_O_2_ is a key element of the innate immune system of mammals in vivo and also have the function of the endosomal compartment to inactivate intracellular pathogens [19]. Importantly, H_2_O_2_ has also been used to detoxify pertussis toxin in infants up to 18 months of age [20]. Compared with commonly used formaldehyde and βPL inactivators, H_2_O_2_ inactivated vaccines are safe and effective[21].

Cytokines are proteins with multiple functions that play a key role in the body's immune response process. Th1 cells can secrete cytokines such as IFN-γ and TNF-β, which mediate the cellular immune response in the body. Th2 cells can secrete cytokines such as IL-4 and IL-10 and can participate in the response process of humoral immunity [22]. IFN-γ can up-regulate the presentation of MHC-II antigen to enhance the activity of CD4^+^ T cells. IFN-γ can also up-regulate the presentation of the MHC class I antigen to increase the sensitivity of CTLs to viral antigens [23]. IL-4 can stimulate the activation of B cells and promote the proliferation of T cells, playing a key role in humoral immunity [24]. IFN-γ and IL-4 are commonly used to detect Th1 and Th2 immune responses [22].

This study, we established an H_2_O_2_ inactivated BVDV method and prepared H_2_O_2_ inactivated BVDV vaccine. After immunizing the mice, we used standardized indirect enzyme-linked immunosorbent assay (ELISA) to detect the levels of BVDV-specific immunoglobulin G (IgG) antibodies for by several ELISA kits for cytokines IFN-γ and IL-4 in the mouse serum. The neutralizing antibody test detects the level of BVDV neutralizing antibody in the serum. Compared with the formaldehyde inactivated BVDV vaccine, we found that the H_2_O_2_ inactivated BVDV vaccine can produce higher specific IgG antibodies. These results indicate that we describe the evaluation of the immune the effect of H_2_O_2_ inactivated BVDV vaccine.

## Materials and Method

### Animal

Thirty female Kunming mice with a 5-week-old age of about 18 g were purchased from the Experimental Animal Center of Xinjiang Medical University. These mice were used for vaccine immunogenicity analysis. All mice are raised in individually ventilated cages (IVC) and can be freely fed. All mice were killed by cervical dislocation after the experiment.

### Virus and commercial vaccine

The MDBK cell line (Free of BVDV and anti-BVDV antibodies) used in this experiment was purchased from the Cell Bank of the Chinese Academy of Sciences (Shanghai, China). BVDV standard strain NADL was purchased from the China Veterinary Drug Administration (Beijing, China) and stored at -80℃. Commercial vaccines was purchased from the Tiankang biological (Xinjiang, China), catalogue no. 310013015. Freund's incomplete adjuvant was purchased from the Sigma-Aldrich (St Louis, MO, USA), catalogue no. 08168. Freund's complete adjuvant s purchased from the Sigma-Aldrich (St Louis, MO, USA), catalogue no. S6322.

### BVDV propagation and titration

In this study, we used MDBK cells to propagate and collect BVDV (3 replicates) [25]. DMEM medium (DMEM; Gibco, Grand Island, NY, USA, catalogue no. 31600034) containing 10% Fetal Bovine Serum (FBS; Gibco, Grand Island, NY, USA, catalogue no. 16000–044) was used to culture MDBK cells at 37 ℃, 5% CO_2_ and saturated humidity. When the cell (the serum-free medium was used) fusion degree reached 90%, cells were infected with multiplicity of infection (MOI) of 1:10 BVDV NADL. After virus adsorption, DMEM medium containing 2% FBS was used. When the cytopathic rate reached 80%, the venom was collected by repeated freezing and thawing. Then the TCID50 of the virus was calculated according to the Reed-Muench method, the calculation formula: Distance ratio = (percentage of lesion rate higher than 50%-50%)/ (percentage of lesion rate higher than 50%-percentage of lesion rate lower than 50%); lgTCID_50_ = distance ratio × difference between logarithm of dilution + logarithm of dilution higher than 50% lesion rate.

### BVDV inactivation with H_2_O_2_ and innocuity testing

In this experiment, the bovine viral diarrhea virus (BVDV, NADL strain) titer was measured with TCID_50_ before inactivation. BVDV solution (10^6.6^ TCID_50_/0.1 ml) were mixed with different concentrations (7%, 5%, 3%, 2%, 1%, 0.5%, 0.3%, 0.1%) of 30% hydrogen peroxide (H_2_O_2_) solution (Sigma-Aldrich, St Louis, MO, USA, catalogue no. USA, 822,287) and warmed in a water bath at different temperatures (22℃, 27℃, 32℃, 37℃, 42℃) and for different times (2 h, 4 h, 6 h, 8 h). Next, 5 μl of 1 mg/ml catalase solution (Solarbio, Beijing, China, catalogue no. C8071) were added to it and incubated at 37 ℃for 10 min to remove residual H_2_O_2_.

The inactivated BVDV was used to infect MDBK cells, and the virus inactivation effect was detected by PCR and gel electrophoresis (3 repetitions per group). TRIzol (Invitrogen, CA, USA, catalogue no. 15596–026) was used to extract viral ribonucleic acid (RNA) from each group of cells. PrimScript™ RT reagent Kit (Takara, Dalian, China, catalogue no. RR037A) to reverse transcribe RNA into complementary deoxyribonucleic acid (cDNA). Primer5.0 software was used to design polymerase chain reaction (PCR) primers according to the sequence of BVDV NADL (NC_001461) 5'UTR region in the GenBank database. The primer sequences are 5’-AGCCATGCCCTTAGTAGGACT-3’ (Forward) and 5’-ACTCCATGTGCCATGTACA-3’ (Reverse). PCR was performed under the following thermocycling conditions: initial denaturation at 95℃ for 5 min, followed by 35 cycles of 95℃ for 20 s, 58℃ for 20 s, and 72℃ for 20 s, using an Eppendorf PCR platform. Detection of PCR production by 2% agarose gel electrophoresis.

### Vaccine preparation

We used 3% H_2_O_2_ to inactivate NADL strain (10^6.6^ TCID_50_/0.1 ml) at 27 ℃ for 2 h to prepare the vaccine for animal experiment. MDBK cells were used to test the safety of virus inactivation.

### Experimental design in mice

We used H_2_O_2_ to inactivate NADL strain (10^6.6^ TCID_50_/0.1 ml) under optimal inactivation conditions to prepare the vaccine for animal experiment. Randomly divided 30 female Kunming mice into 3 groups, each with 10 mice, a total of 70 days of experiments were conducted. Mice in the experimental group were immunized with H_2_O_2_ inactivated vaccine (H_2_O_2_ group, *n* = 10) and formaldehyde inactivated vaccine (Formaldehyde group, *n* = 10,) [26]. In the control group, mice were immunized with equal volume sterilized phosphate buffer saline (PBS) solution and adjuvant after emulsification instead of H_2_O_2_ inactivated BVDV vaccine (NC, *n* = 10). For the first immunization, the inactivated venom was emulsified with Freund's complete adjuvant and 100 μl (10^6.6^ TCID_50_/0.1 ml) was subcutaneous injected into each mouse. After 14 days, the mice were immunized again with the inactivated venom and Freund's incomplete adjuvant.

### BVDV IgG, IFN-γ and IL-4 determination

The blood of all mice was collected before immunization. From the 14th day after immunization, serum from mice in the experimental group and the PBS negative control group were collected weekly. 3 mice were randomly selected from each group, and an indirect enzyme-linked immunosorbent assay (ELISA) test was used to detect the level of BVDV-specific immunoglobulin G (IgG) antibody in the serum. BVDV solution was coated at a dilution of 1:16. The proportion of serum dilution 1:500 to remove the primary antibody and 1:500 to remove the secondary antibody. and applied to BVDV-coated plates. HRP-labeled goat anti-mouse IgG (Abcam, Cambridge, MA, catalogue no. ab6789) was used to detect the level of bound antibodies. The microplate reader detects the OD_450_ value and calculated the P/N value. Mouse IFN-γ ELISA kit (Soleibao Technology Co., Ltd., Beijing, China, catalogue no. SEKM-0031), mouse IL-4 ELISA kit (Soleibao Technology Co., Ltd., Beijing, China, catalogue no. SEKM-0005) weredetected the contents of Th1 type cytokine IFN-γ and Th2 type cytokine IL-4 in serum samples following manufacture instructions.

### Determination of virus neutralising antibodies

On the 28th and 42nd days after the second immunization, each group of mice were subjected to orbital blood collection with capillary glass tubes (*n* = 3), and heat-inactivated in a constant temperature water bath at 56℃ for 30 min. The concentration of serum samples to be tested is diluted to 2^1^–2^8^ by DMEM cell culture medium containing 2% FBS. BVDV NADL strain (200 TCID_50_/100 μl) was diluted with equal volume serum of 50 μl and incubated for 90 min. MDBK cells were plated on 96-well cell culture plates at a cell seeding density of 2 × 10^5^ cells ml^−1^ and cultured for 24 h. Then added the mixture of serum and BVDV NADL strain and cultured for 72 h to observe the degree of cytopathic changes. The titer of neutralizing antibody to H_2_O_2_ inactivated vaccine can be calculated by diluting mouse serum to different multiples and then mixing it with virus to observe the minimum dilution of cytopathy.

### Statistical analysis

The data were analyzed using SPSS19.0 (IBM Corp., NY, USA). Groups were compared using mixed ANOVA followed by Dunnett's post-hoc test. A *P*-value < 0.05 (* *P* ≤ 0.05) was considered to be significant difference, a *P*-value < 0.01 (** *P* ≤ 0.01) was considered to be extremely significant.

## Results

### virus cytopathogenicity and infectivity

**Fig. 1:**
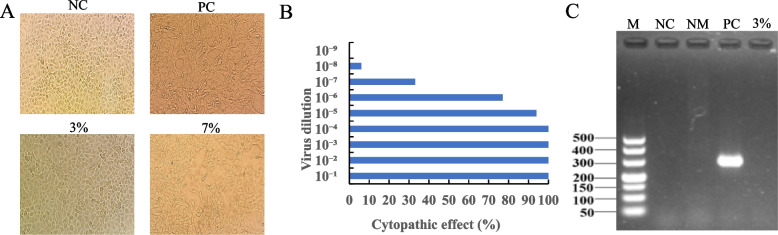
MDBK cells were infected with H_2_O_2_ inactivated BVDV NADL. **A** Cell status after infection with BVDV. NC, normal MDBK cells; PC, positive control; 3%, MDBK cells were infected with BVDV inactivated by 3% H_2_O_2_ at 27℃ for 2 h; 7%, MDBK cells were inoculated with BVDV inactivated by 7% H_2_O_2_ at 27℃ for 2 h. The photo was taken with an inverted microscope from Shanghai Optical Instrument Factory, with a magnification of 400 × . **B** TCID 50 results of MDBK cells inoculated with BVDV NADL. **C** PCR result of 5’-UTR gene in cell culture. M, DL 500 DNA Marker; NC, negative control; NM, normal MDBK cell; PC, positive control; 3%, cells inoculated with H_2_O_2_ inactivated virus

Bovine viral the number of holes of each dilution lesion diarrhea virus (BVDV) solution was inoculated on normal-growing MDBK cells and observed through an inverted microscope. After infection with BVDV, the edge of the cell is blurred, and the phenomenon of stretching and deformation occurred (Fig. 1A). Observed the cell lesions and recorded the number of holes of each dilution lesion. The TCID_50_ of the virus calculated by the Reed-Muench method is 10^6.6^ TCID_50_/0.1 ml (Fig. 1B).

### H_2_O_2_ inactivated the BVDV

After culturing MDBK cells with different concentrations of H2O2 inactivated BVDV (106.6 TCID50/0.1 ml) for 4 days, it was observed through an inverted microscope that the cells at the 3 inactivation concentrations of 3%, 2%, and 1% did not show cytopathic conditions. But the morphology cells in the 7% and 5% inactivated groups changed, and some cells fell off (Fig. 1A). BVDV solution was inactivated by 3% H_2_O_2_ at 27℃ for 2 h and then inoculated into well-growing monolayer MDBK cells. After three generations of blind transmission, no cytopathic effect was found compared with the control group (Fig. 1A). The total ribonucleic acid (RNA) extracted from each group of cells was subjected to PCR identification after reverse transcription. The results showed that no viral nucleic acid was detected in the cell culture (Fig. 1C), which indicated that the BVDV was completely inactivated and could be used for later vaccine preparation.

### BVDV-specific IgG antibody levels

**Fig. 2:**
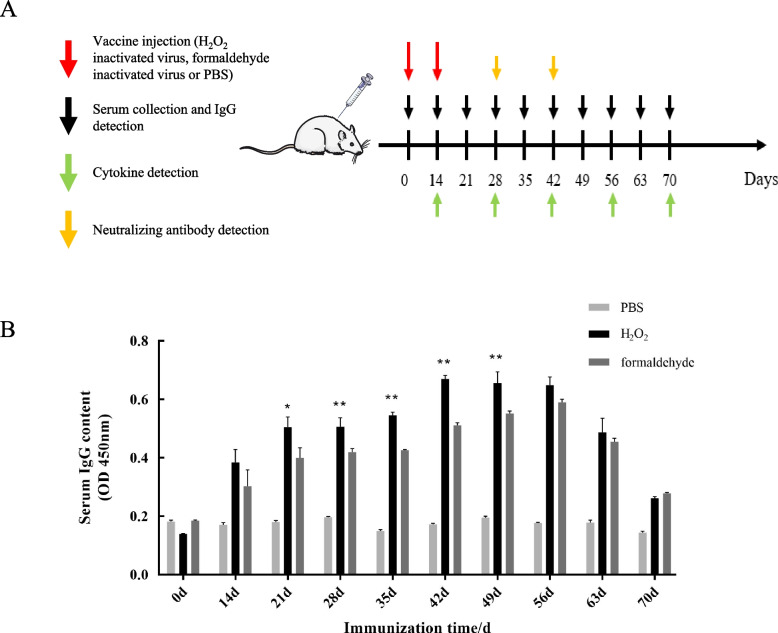
Detection of antibody levels in immunized mice. **A** Inactivated virus vaccine experiment design diagram. **B** Detection of serum BVDV-specific IgG antibody levels in immunized mice. Each sample was tested 3 times independently, and the test data was plotted with GraphPad prism software, *P* ≤ 0.05, the difference was significant; *P* ≤ 0.01, the difference was extremely significant (* *P* ≤ 0.05, ** *P* ≤ 0.01)

Mice were vaccinated with the H_2_O_2_ group, formaldehyde group and NC group vaccine to evaluate the humoral immunity induced by the inactivated vaccine (Fig. 2A). On the first day, there was no significant difference in serum antibodies between the H_2_O_2_ inactivated vaccine group and the formaldehyde inactivated vaccine group compared with the phosphate buffer saline (PBS) control group (*P* > 0.05). On the 14th to 70th day, the difference between the two groups of inactivated vaccine groups and the PBS control group was extremely significant (*P* < 0.01). The serum-specific antibodies in the H_2_O_2_ group were significantly higher than that in the formaldehyde group on the 21st to 28th day (*P* < 0.05). From 35 to 49d, the level of BVDV-specific immunoglobulin G (IgG) antibody in the serum of mice immunized with the H_2_O_2_ inactivation group was significantly higher than that in the formaldehyde group (*P* < 0.01). After 56 days, there was no significant difference in antibody levels between the two experimental groups (*P* > 0.05) (Fig. 2B). Therefore, the two vaccines prepared with different inactivating agents can make the immunized mice produce an obvious humoral immune response, and the antibody level raised with the immunization time.

The level of inducing humoral immunity is an important indicator for evaluating the immune effect of inactivated vaccines. In this experiment, the levels of specific immunoglobulin G (IgG) antibodies in the serum of the two vaccines were increased after the mice were inoculated. The BVDV-specific antibody in the serum of mice in the H_2_O_2_ inactivated group reached the level with a positive/negative (P/N) value of 2.18 on the 14th day after the first immunization. In the formaldehyde-inactivated group, the BVDV-specific IgG antibody in the mouse serum reached a level on the 21st day (P/N value 2.27). In addition, the serum-specific antibodies in the H_2_O_2_ group were significantly higher than those in the formaldehyde group in the early stage after immunization (*P* < 0.05). After 56 days, there was no significant difference in antibody levels between the two experimental groups (*P* > 0.05). Therefore, the results showed that the two vaccines prepared with different inactivating agents can make the immunized mice produce an obvious humoral immune response, and the antibody level raised with the immunization time. However, the H_2_O_2_ inactivated group can induce higher levels of BVDV-specific IgG antibody levels in a shorter time (*P* < 0.05), which indicates that the vaccine prepared by this method is superior to traditional formaldehyde vaccines in inducing specific IgG antibodies.

### Serum specific cytokine detection

**Fig. 3:**
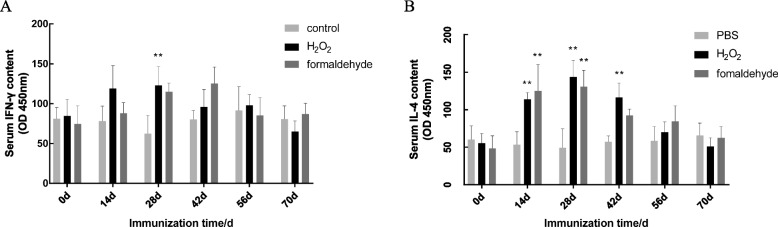
Changes in cytokine levels in immunized mice. **A** Changes in IFN-γ secretion levels in immunized mice. **B** Changes in IL-4 secretion levels in immunized mice. Each sample was tested 3 times independently, and the test data was plotted with GraphPad prism software, *P* ≤ 0.05, the difference was significant; *P* ≤ 0.01, the difference was extremely significant (* *P* ≤ 0.05, ** *P* ≤ 0.01)

In order to evaluate the level of cellular immunity produced by the inactivated vaccine, the Mouse IFN-γ ELISA kit and mouse IL-4 ELISA kit was used to quantitatively analyze the changes of IFN-γ and IL-4 in the serum of each group of immunized mice. The serum IFN-γ level of mice in the H_2_O_2_ group gradually increased from 14 to 28 d after immunization, and reached a peak on the 28th day (Fig. 3A). The serum IFN-γ content of the formaldehyde group was gradually increased from day 14 until the peak on day 42, which gradually decreased to the pre-immune level on day 56 (Fig. 3A). The formaldehyde group was lower than the H_2_O_2_ group on the 14th and 28th days, but the difference was not significant (*P* > 0.05).

The level of IL-4 in the serum of mice in H_2_O_2_ group increased from the 14th day after the first immunization to the peak on the 28th day, and was significantly higher than the PBS group (*P* < 0.05). It began to decrease after the 42nd day, and basically returned to the pre-immune level on the 70th day (Fig. 3B). The level of IL-4 in the serum of mice in the formaldehyde group also began to increase on the 14th day, and reached a peak on the 28th day, which was significantly higher than that of the PBS group (*P* < 0.05). From 14 to 42 d after immunization, the level of IL-4 in the H_2_O_2_ inactivated group was higher than that in the formaldehyde group, but the difference was not significant (*P* > 0.05) (Table 1).

**Table 1:** Effects of different vaccines on IgG, IFN-γ and IL-4 contents in serum of mice at different time points

	Group				*P*-value		
Item	NC	H_2_O_2_	Formaldehyde	SEM	Group	Time	Group × Time
**IgG**							
day0	0.18	0.14	0.18	0.00	< 0.01	< 0.01	< 0.01
day14	0.17^a^	0.38^b^	0.30^b^	0.02			
day21	0.18^a^	0.50^b^	0.40^c^	0.01			
day28	0.20^a^	0.51^b^	0.42^c^	0.01			
day35	0.15^a^	0.55^b^	0.43^b^	0.01			
day42	0.17^a^	0.67^b^	0.51^c^	0.01			
day49	0.20^a^	0.66^b^	0.55^c^	0.01			
day56	0.18^a^	0.65^b^	0.59^b^	0.01			
day63	0.18^a^	0.49^b^	0.46^b^	0.02			
day70	0.14^a^	0.26^b^	0.28^b^	0.01			
**IFN-γ**							
day0	81.04	84.69	74.69	11.30	0.142	0.036	< 0.01
day14	78.17	119.13	88.06	12.24			
day28	62.50	123.16	115.19	11.50			
day42	80.32	96.24	125.52	10.62			
day56	91.73	98.24	85.22	13.17			
day70	80.83	65.12	87.07	8.37			
**IL-4**							
day0	60.19	55.43	48.35	9.35	< 0.01	< 0.01	< 0.01
day14	53.50^a^	114.00b	124.98^bc^	13.39			
day28	49.32^a^	143.80^b^	130.87^bc^	13.33			
day42	57.35^a^	116.44^b^	92.39^bc^	7.47			
day56	58.64	69.96	84.49	10.46			
day70	65.63	50.96	62.51	8.44			

^a,^^b,c^ Means in the same row with different superscripts are significantly different (*P* ≤ 0.05). Ifn-γ and IL-4 concentration units are pg/ml

### Neutralizing antibody detection

A neutralization test is used to detect whether mice can produce neutralizing antibodies against BVDV after vaccination. The results showed that on the 28th day after the first immunization, the average neutralizing antibody titer in the H_2_O_2_ inactivated vaccine group was 6 Nu while the average antibody titer produced by the formaldehyde inactivated vaccine was 6 Nu. There was no neutralizing antibody produced in the PBS group (Table 2). On the 42nd day after the first immunization showed that the average antibody titer in the H_2_O_2_ inactivated vaccine group was 12 Nu. The average value of the formaldehyde inactivation group was 7 Nu. The PBS control group did not produce neutralizing antibodies. At this time, the antibody titer of the H_2_O_2_ inactivated vaccine group was consistent with the formaldehyde inactivated vaccine group (Table 2). With the increase in immunization time, the neutralizing antibody titer of BVDV in the H_2_O_2_ inactivated vaccine group gradually increased, and the neutralizing antibody titer could reach 12 Nu in the 6th week.

**Table 2:** Test results of BVDV neutralizing antibody titer in each group after immunization

	Mouse	Neutralizing antibody titer (28th day)(Nu)	Neutralizing antibody titer (42th day) (Nu)
H_2_O_2_ inactivated BVDV vaccine	*n* = 3	6	12
Formaldehyde inactivated BVDV	*n* = 3	6	7
PBS solution	*n* = 3	0	0

## Discussion

The standard method for preparing inactivated vaccines is to inactivate pathogens with formaldehyde or β-propiolactone (βPL) [14]. However, due to the hazards of formaldehyde or βPL, a safe hydrogen peroxide (H_2_O_2_) inactivation method was selected for this test. H_2_O_2_ inactivated virus had been found to effectively promote cellular immune response with less cytotoxicity to cells [27]. Amanna et al. found that the yellow fever virus (YFV) -17D strain and West Nile virus (WNV)-NY99 strain of the Flaviviridae family can be completely inactivated in 2 h under the conditions of 3% H_2_O_2_ and 20℃-24℃ [26]. This experiment used H_2_O_2_ as the inactivator to explore the conditions for H_2_O_2_ to completely inactivate the bovine viral diarrhea virus (BVDV) NADL strain. Through verification at the cell level and molecular level, Cells can survive for 4 days at 27℃, 37℃ and 42℃ under virus inactivation conditions, but 37℃ and 42℃ are a large and small number of adherent cells, so we chose 27℃. In addition, we found that the inactivation time had no effect on virus inactivation. Therefore, we chose 2 h H_2_O_2_ with the shortest time. 1%, 2% and 3% concentration can inactivate BVDV. In order to ensure better inactivation effect, we used 3% concentration H_2_O_2_. So we chose condition 3% H_2_O_2_ at 27℃ for 2 h, can not only inactivate viruses, but also bacteria [28]. bacterial spores [29]. and parasites [30]. It has a wide range of uses in the field of vaccine preparation.

IFN-γ is secreted by Th1 type cells and mediates cellular immune response; IL-4 is secreted by Th2 type cells and mediates humoral immunity. The quantitative results of the cytokines in this study showed that the H_2_O_2_ inactivated BVDV vaccine can induce the same level of the cellular immune response as the traditional formaldehyde inactivated vaccine and maintain it for about 42 days. In previous studies, Pinto et al. also demonstrated that H_2_O_2_ was used to inactivate the West Nile virus [31]. which has high homology with BVDV. After immunizing the mice, it produced a strong cellular immune response and prevented the lethal West Nile virus infection in mature mice. These all show the reliability of the method. There was some fluctuation in IFN-γ data, possibly because 3 mice were randomly selected to collect serum for each measurement, which was caused by differences between individuals.

Most licensed vaccines provide protective immunity by inducing neutralizing antibodies [32]. and it had been established many years ago that the neutralizing antibodies induced by the yellow fever vaccine are closely related to the protective effect [33]. Neutralizing antibodies are special antibodies that can specifically bind to bacterial toxins, pathogens (such as viruses), and their products to neutralize them. Research has shown that inactivated polio vaccine (IPV) can stimulate serum antibodies and has a protective effect with a neutralization degree of 1/8 (or even 1/4) [34]. We detected that in the H_2_O_2_ inactivated vaccine group, the neutralizing antibody titer can reach 1:32 in the 6th week, indicating that the vaccine inactivated by this method can protect against BVDV infection. The mean neutralization titer of the formaldehyde group was higher than that of the H2O2 group on day 28, but the opposite was true on day 42, possibly because three mice were randomly selected to collect serum for each measurement, which was caused by differences between individuals. The comparable neutralization antibody from two group shows that H_2_O_2_ may be used as a new method to prepare BVDV inactivated vaccine. However, it must be recognized that the immunizations used in this study were performed in mice rather than cattle, it has certain limitations, which may offer limited advice for the practical application of the vaccine. The small number of experimental animals and the lack the challenging experiments at the individual level are the main limitations of this study. In addition, the differences in the immune effects of different vaccination doses and vaccination methods were not explored. In further research, we will explore the optimal dose and method of vaccination and the immune effect of the vaccine in cattle to further evaluate the possibility of H_2_O_2_ inactivated BVDV vaccine in practical application.

## Conclusions

In conclusion, hydrogen peroxide (H_2_O_2_) as an inactivator has become a research hotspot in the field of vaccines. In this study, H_2_O_2_ inactivated vaccine was prepared using the bovine viral diarrhea virus (BVDV) NADL strain. It can not only stimulate mice to produce high levels of BVDV-specific immunoglobulin G (IgG) antibodies, but also enable the body to produce neutralizing antibodies against BVDV and induce induce IFN-γ and IL-4 response. Finally, it can provide a method and potential solution for the further development of efficient and safe BVDV vaccines.

### Supplementary Information

**Additional file 1**

## Data Availability

All the data are also available from the corresponding author on reasonable request.
