# Supplementary figures and images for: Efficacy of H2O2 inactivated bovine virus diarrhoea virus (BVDV) type 1 vaccine in mice
Source: BMC Vet Res. 2024 Feb 3;20:43. doi: 10.1186/s12917-024-03897-0 (PMC10837870; doi:10.1186/s12917-024-03897-0)

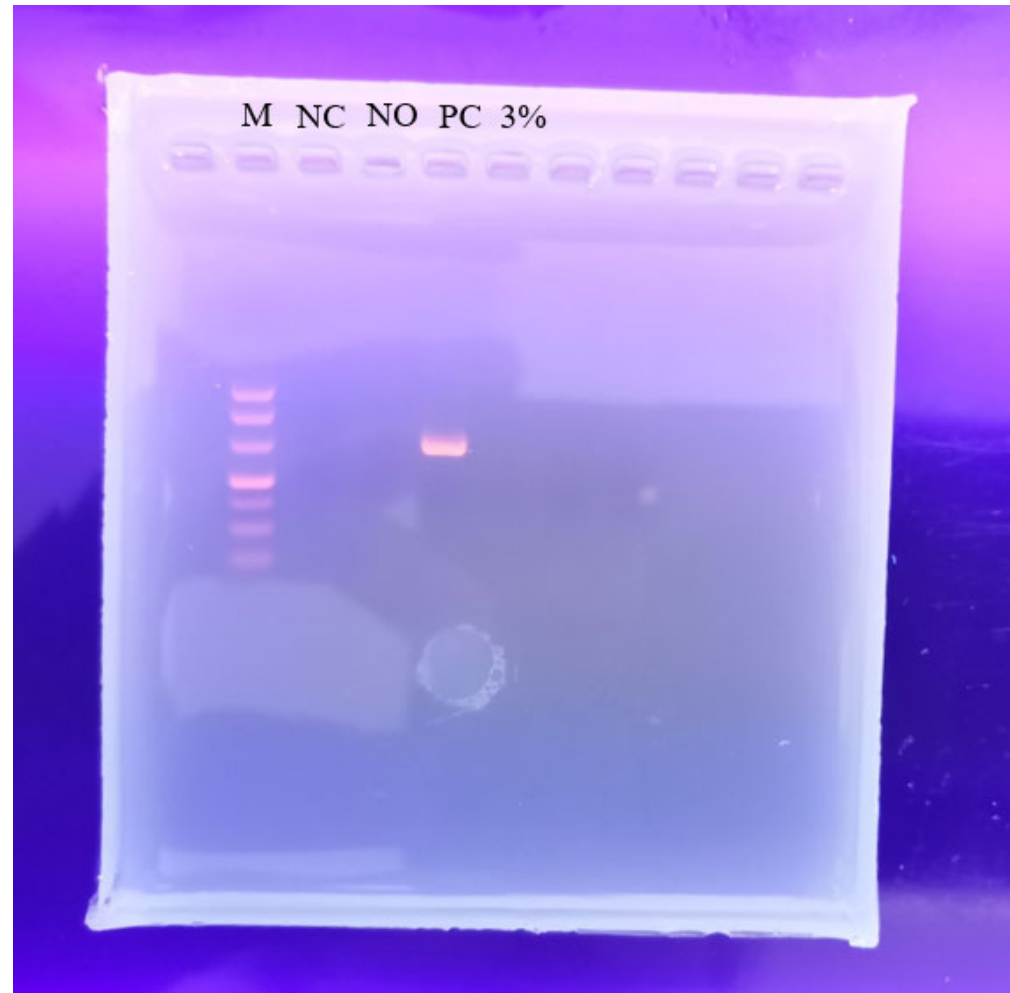

Supplement: Supplementary file 1 — Additional file 1 [file 12917_2024_3897_MOESM1_ESM.pdf]
